# Behavioral innovations in the management of obesity and overweight using the COM-B model: Vladimíra Timková

**DOI:** 10.1093/eurpub/ckac131.352

**Published:** 2022-10-25

**Authors:** V Timkova, D Minarikova, Z Katreniakova, P Mikula, A Husivargova, I Nagyova

**Affiliations:** 1 Department of Social and Behavioural Medicine, Faculty of Medicine, PJ Safarik University in Kosice, Košice, Slovakia; 2 Organisation and Management of Pharmacy, Faculty of Pharmacy, Comenius University in Bratislava, Bratislava, Slovakia

## Abstract

**Background:**

Increasing overweight and obesity rates represent one of the global health challenges in the 21st century. Obesity is a gateway to many non-communicable diseases and shorter life expectancy. Understanding individual capability, opportunity, and motivation for health behavior change (COM-B) may help to develop effective public health interventions. However, so far, only a very few studies examined interventions based on behavior change theories. Thus, we aimed to assess the potential facilitators and barriers to behavior change in weight management using the COM-B model.

**Methods:**

We included 146 people with overweight and obesity (66.7% female; mean age 46.62±14.51 years; body mass index 32.46±6.51 kg/m2) from primary care settings. All participants completed the Brief Measure of Behavior Change (COM-B), the General Self-Efficacy Scale (GSE), the Rosenberg Self-esteem Scale (RSE), and the Self-Rated Heath scale (SRH). Multiple linear regression analyses were used to analyze the data.

**Results:**

In univariate analyses COM-B domains were positively associated with female gender, living with a partner, lower body mass index, and higher SRH. However, the associations between sociodemographic and clinical variables and COM-B domains were no longer significant when self-esteem and general self-efficacy were added to the multivariate regression models. Self-efficacy was associated with reflective motivation (β: 0.53; p < 0.001), physical capability (β: 0.43; p < 0.001), and psychological capability (β: 0.44; p < 0.001) the most significantly. Self-esteem was strongly associated with physical capability (β: 0.41; p < 0.01). Total explained variances in the final models varied from 17.7% to 25.0%.

**Conclusions:**

Personal resources may play a significant role in weight management and should be included in tailored public health interventions (Grant support: VEGA: 1/0748/22).

**Key messages:**

• Interventions focused on the enhancement of personal resources may improve weight management.

• Behavioral and cultural aspects should be considered when designing effective public health interventions.

